# ^18^F-FDG-PET/CT-based deep learning model for fully automated prediction of pathological grading for pancreatic ductal adenocarcinoma before surgery

**DOI:** 10.1186/s13550-023-00985-4

**Published:** 2023-05-25

**Authors:** Gong Zhang, Chengkai Bao, Yanzhe Liu, Zizheng Wang, Lei Du, Yue Zhang, Fei Wang, Baixuan Xu, S. Kevin Zhou, Rong Liu

**Affiliations:** 1 Medical School of Chinese PLA, Beijing, China; 2 Faculty of Hepato-Biliary-Pancreatic Surgery, The First Medical Center of Chinese People’s Liberation Army (PLA) General Hospital, 28 Fuxing Road, Beijing, 100853 China; 3 School of Biomedical Engineering, Division of Life Sciences and Medicine, University of Science and Technology of China, Hefei, Anhui China; 4 Senior Department of Hepatology, The Fifth Medical Center of Chinese People’s Liberation Army (PLA) General Hospital, Beijing, China; 5 Department of Nuclear Medicine, The First Medical Center of Chinese People’s Liberation Army (PLA) General Hospital, Beijing, China; 6 Suzhou Institute for Advanced Research, University of Science and Technology of China, Suzhou, Jiangsu China

**Keywords:** Deep learning, Pancreatic cancer, PET/CT, Pathological grading, Prediction model

## Abstract

**Background:**

The determination of pathological grading has a guiding significance for the treatment of pancreatic ductal adenocarcinoma (PDAC) patients. However, there is a lack of an accurate and safe method to obtain pathological grading before surgery. The aim of this study is to develop a deep learning (DL) model based on ^18^F-fluorodeoxyglucose-positron emission tomography/computed tomography (^18^F-FDG-PET/CT) for a fully automatic prediction of preoperative pathological grading of pancreatic cancer.

**Methods:**

A total of 370 PDAC patients from January 2016 to September 2021 were collected retrospectively. All patients underwent ^18^F-FDG-PET/CT examination before surgery and obtained pathological results after surgery. A DL model for pancreatic cancer lesion segmentation was first developed using 100 of these cases and applied to the remaining cases to obtain lesion regions. After that, all patients were divided into training set, validation set, and test set according to the ratio of 5:1:1. A predictive model of pancreatic cancer pathological grade was developed using the features computed from the lesion regions obtained by the lesion segmentation model and key clinical characteristics of the patients. Finally, the stability of the model was verified by sevenfold cross-validation.

**Results:**

The Dice score of the developed PET/CT-based tumor segmentation model for PDAC was 0.89. The area under curve (AUC) of the PET/CT-based DL model developed on the basis of the segmentation model was 0.74, with an accuracy, sensitivity, and specificity of 0.72, 0.73, and 0.72, respectively. After integrating key clinical data, the AUC of the model improved to 0.77, with its accuracy, sensitivity, and specificity boosted to 0.75, 0.77, and 0.73, respectively.

**Conclusion:**

To the best of our knowledge, this is the first deep learning model to end-to-end predict the pathological grading of PDAC in a fully automatic manner, which is expected to improve clinical decision-making.

**Supplementary Information:**

The online version contains supplementary material available at 10.1186/s13550-023-00985-4.

## Background

Pancreatic cancer is a common malignancy and is the fourth most deadly cancer in the world [1], killing approximately 480,000 people worldwide each year. In the next decade, pancreatic cancer is likely to become the second leading cause of death [2]. Surgery is currently the only treatment that may cure pancreatic cancer. Statistics show [3] that 10% of patients with indications for surgical resection have a 5-year survival rate of 24.6% (and 2.9%) for patients who have (and have not) undergone pancreatic ductal adenocarcinoma (PDAC) resection in Stage I.

Predicting pathological grading of PDAC is an important part of the diagnosis and treatment of PDAC. Pathological differentiation of PDAC helps assess the extent, depth, and metastatic status of pancreatic cancer, which is an important basis for determining the best treatment plan and predicting prognosis. It plays an important role in guiding surgery and corresponding adjuvant therapy for precise individualized treatment. A study by Golan et al. showed that well-differentiated PDAC was associated with long-term survival after surgery [4]. In contrast, poor differentiation is an independent prognostic factor affecting overall survival [5]. For patients with poorly differentiated PDAC, neoadjuvant therapy may provide longer survival than direct surgery [6–8]. The only method currently available to determine PDAC grading preoperatively is ultrasound or computed tomography (CT)-guided puncture biopsy. Tumor tissue columns obtained in this manner do not reliably reflect the structural features of the entire lesion due to their high heterogeneity [9]. A study by Larghi et al. showed that the preoperative grading of Endoscopic ultrasound-guided fine-needle biopsy (EUS-FNB) had an accuracy of 56%, sensitivity of 41%, and specificity of 78% [10]. Therefore, a safe and accurate preoperative method for determining the degree of differentiation of PDAC is needed.

The determination of the pathological grading of PDAC relies on pathological slices as pathological examination is the gold standard for diagnosing the disease. However, since pathological tissue is obtained through invasive puncture or surgery, the pathological results have a pronounced lag. At present, the diagnosis of PDAC is frequently conducted through noninvasive medical imaging techniques, including CT scans, magnetic resonance imaging (MRI), and positron emission tomography (PET). These methods enable quicker diagnostic results. However, due to low image resolution, the information in medical images is not as clear as that in pathological slices. Also, the variations in equipment and operator lead to unstable imaging results, or at least not as stable as pathological examination. Therefore, how to automatically predict the pathological grading of PDAC through imaging data is a challenging task. In this paper, we attempt to bridge this gap.

At present, some researchers have made some useful explorations. Vincent et al. [11] found an inverse correlation between apparent diffusion coefficient (ADC) and SUV while only ADCmin was significantly correlated with tumor grade in PDAC patients. Xing et al. [12] used machine learning to establish a predictive model based on PET/CT imaging features, which divided PDAC patients into grade 1 and Grade 2/3 groups, with an AUC of 0.994 in the training set and 0.921 in the validation set. Deep learning is a machine learning method that automatically learns features and classifies through the design and use of multi-layer networks [13, 14]. In recent years, deep learning has been widely applied in medical image analysis, including PET/CT image analysis [15]. PET/CT is a whole-body functional imaging examination, which reflects the malignancy or benignity of lesions through the metabolic activity of cells. Wang et al. [16] studied the use of deep learning models to segment lung cancer in PET/CT images and achieved a high accuracy [12]. Chao et al. [17] used a dual-energy CT-based deep learning radiomics model to classify PDAC's lymph node metastasis (LNM) status, and the model's AUC was 0.87.

These studies indicate that deep learning models based on PET/CT have a high accuracy and sensitivity. However, current research on pathological grading of PDAC is still limited. Therefore, our goal is to establish a fully automated deep learning model based on ^18^F-fluorodeoxyglucose (^18^F-FDG)-PET/CT for predicting preoperative pathological grading of PDAC.

## Materials and methods

**Fig. 1:**
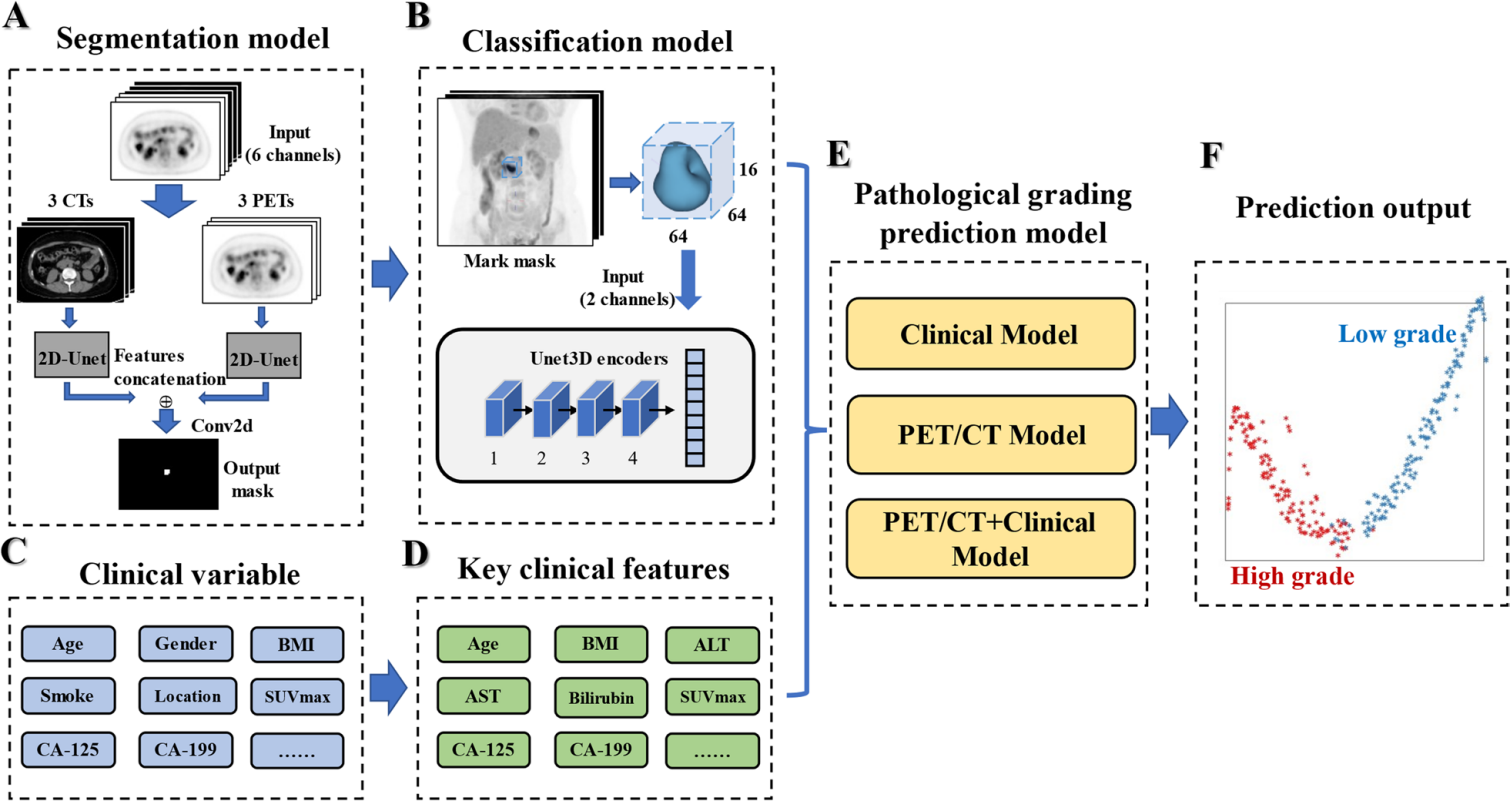
The workflow of DL model based on PET/CT for pathological grading of patients with pancreatic ductal adenocarcinoma (PDAC)

Figure 1 presents the schematic workflow of the proposed deep learning (DL) model based on PET/CT for pathological grading of patients with PDAC, which consists of multiple processing stages. Below, we elaborate the details related to the workflow, starting with study population, image labeling, model construction, and finally model testing.

### Study population

Patients who underwent pancreatic surgery at the PLA General Hospital from January 2016 to September 2021 and obtained pathological confirmation of PDAC were collected and included in the study according to the inclusion and exclusion criteria, and 370 patients were finally included.

Inclusion criteria: (i) PDAC was pathologically confirmed by radical pancreatic resection; (ii) PDAC was confirmed by pathological biopsy of non-radical pancreatic surgery; (iii) PET/CT of the pancreas was performed within 1 month before surgery. Exclusion criteria: (i) patients had adjuvant treatment such as radiotherapy, chemotherapy and intervention before surgery; (ii) PET/CT images were of poor quality (tumor and borders could not be distinguished with the naked eye or there were artifacts interfering) and could not be used to analyze patients; (iii) other malignant tumors were combined; (iv) pathological findings and images could not correspond. Clinical data such as a patient’s age, gender, preoperative CA199 level, tumor location, tumor size (long and short diameter) on PET/CT images, and SUVmax values were also collected.

### PET-CT image labeling process

Supplementary Method 1.1 provides detailed information about the PET/CT scanning protocol. The regions of abnormal ^18^F-FDG uptake on PET and density abnormality on CT are localized as the lesion region as follows. After the PET/CT image fusion is completed, two experienced PET/CT diagnostic physicians use 3D slicers (version 5.1.0, https://www.slicer.org) software with a threshold of 40% SUVmax to draw out the ROI (Region of Interest) of the target lesion, and all discrepancies are confirmed through discussion. All images were analyzed by two senior nuclear medicine experts (each with over 5 years of experience in PET interpretation). The analysis included aspects such as tumor lesion location, size, standard uptake value (SUVmax), relationship with surrounding tissues, liver mean standard uptake value (SUVmean), SUVR (tumor-to-normal liver standard uptake value ratio, SUVmax of the tumor /SUVmean of the normal liver parenchyma), presence of lymph node metastasis, presence of distant metastasis, and observations under various sequences.

The patients' ^18^F-FDG PET/CT scans were obtained from three different machines. Consequently, measurements of metabolic parameters may exhibit variations due to differences in machine design and scintillation detectors [18–20]. We cannot exclude that such differences may have at least in part confounded SUVmax measurements. To address this problem, we retrospectively calculated the mean SUV values of hepatic parenchyma in 370 patients with original PET/CT images (GE Discovery VCT, *n* = 161; Siemens Biography 64 PET/CT, *n* = 166; uMI 510 PET/CT, *n* = 43). To measure normal liver parenchyma activity, 3 non-overlapping spherical 1-cm^3^-sized VOIs were drawn in the normal liver on the axial PET images. There were no significant differences in terms of SUVmean-liver among the 3 PET/CT scanners (GE Discovery VCT, 2.30 ± 0.48 vs. Siemens Biograph 64, 2.28 ± 0.38 vs. uMI 510, 2.35 ± 0.29, respectively; *F* = 0.407, *p* = 0.666, variance analysis).

### Constructing the lesion segmentation model

The whole process of building the deep model for lesion segmentation is shown in Fig. 1A. 100 cases of annotated PET/CT images of pancreatic cancer were input into the segmentation model for training.

The PET-CT images were first pre-processed: (a) Window width and window level (350, 40) were applied to intercept the gray value; (b). Each pair of 3D CT series (512*512*H_CT_) and 3D PET series (of size 96*96*H_PET_, 128*128*H_PET_, 168*168*H_PET,_ 170*170*H_PET_) were uniformly resized to 256*256*H_PET_; (c) For each slice, the gray scale was normalized [0,1]; (d) 3 PET slices and 3 CT slices centered around the corresponding location form a 6-channel input to the model.

Model construction: The 6-channel input has a PET part and a CT part, each fed into a 2D-Unet network branch with no shared parameters. The feature vectors of the two 2D-Unets are then concatenated to pass through convolutions, which output the final lesion segmentation mask (Additional file 1: Fig.S1.). We employed a batch size of 8 and early stopping for choosing the best training step. The learning rate was set to 1 × 10^–5^, and the parameters were updated using the Adam optimiser.

Post-processing the segmentation result: (a) All slices in a patient case were predicted and combined into a complete 3D mask; (b) A pre-trained nnUnet [21] model of organ segmentation was loaded to provide a coarse segmentation of the abdominal organs. The predicted segmentation of nnUnet gave the location of pancreas. It was used to reduce the wrong segmentation in other organs when fused with the pancreatic tumor segmentation results from 2D-Unettumor; (c) Medical image analysis techniques including erosion, expansion, and SUVmax 40% threshold segmentation are further applied to obtain the final lesion segmentation results.

### Building PDAC pathological grade classification models

Due to the low prevalence of pathological samples with extreme pathological differentiation grades in the clinic, the grades with few samples were merged in this study and all samples were set to two predictive labels: low grade or high grade. Highly, moderate-highly, and moderately differentiated pathologies were defined as low grade; undifferentiated, lowly, and moderate-lowly differentiated were defined as high grade (Additional file 1: Fig. S2.). This is similar to the classification method of Wasif and Rochefort et al. [22, 23]

According to the segmentation result, the lesion regions were cropped out of the 3D data of PET, CT, and segmentation Mask, respectively, and three aligned copies of size 64*64*16 (length*width*height) were obtained. The CT data were intercepted with a window width and window level (350, 40) and normalized to [0,1], and the PET data were normalized to [0,1]. The PET/CT, cropped according to segmentation mask, were concatenated in the channel dimension to obtain a tensor of size 2*64*64*16 (number_of_channels*length*width*height). The tensor was fed into a Unet3D-based Encoder to extract image feature vectors as shown in Fig. 1B. The overall network model structure diagram is provided in Additional file 1: Fig. S3.

Cases with clinical data missing ratios greater than 20% were excluded from our study. A total of 21 clinical variables were collected to build predictive models based on clinical experience and literature reports. Subsequently, the individual clinical data were analyzed for significance using the Random Forest method (Additional file 1: Fig.S4.). Eleven important clinical characteristics including age, BMI, SUVmax, ALT, AST, total bilirubin, direct bilirubin, blood glucose, CEA, CA125 and CA199 were kept. Finally, the clinical data feature vectors were extracted using the MLP through the multi-layer perceptron. The part was shown in Fig. 1C, D.

Both image features and clinical data features can be used to obtain prediction results for their respective modalities through the fully connected (FC) layer. To obtain better prediction performance, we replaced the last FC layer with a TMC (Trusted Multi-view Classification) [24] to integrate image features and clinical data features and constructed a PET/CT + Clinical data model. TMC is a new multi-view classification algorithm that dynamically integrates different views at an evidence level to promote classification reliability by considering evidence from each view (Additional file 1: Method 1.2). The learning rate was set to 1 × 10^–5^, and the parameters in the feature extractor were updated using the Adam optimiser.

### Sevenfold cross-validation for model testing

**Fig. 2:**
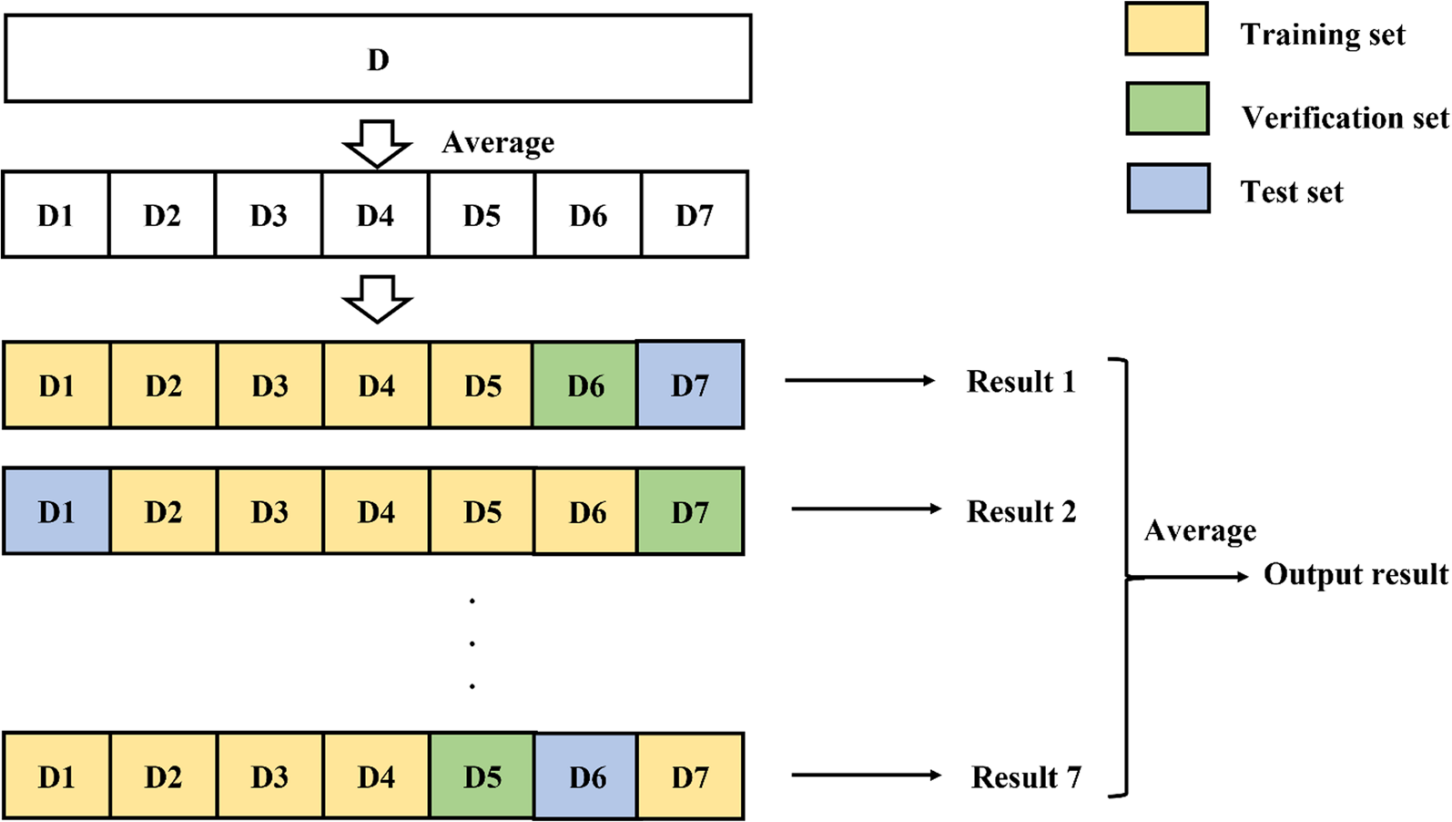
Sevenfold cross-validation model

We used a sevenfold cross-validation to better evaluate the generalization ability of the model. This is shown in Fig. 2. We divided 370 patients into 7 folds, of which 5 folds were the training set for the model in each training round, onefold was the internal validation set and onefold was used as the test set to test the final performance of the model. The next round was trained by changing the order of the training, validation and test folds. The final model is obtained by averaging the results of the 7 folds.

### Statistical analysis

The clinical data were statistically processed using SPSS 22.0 statistical analysis software: normally distributed measures were expressed as *x* ± *s* and comparisons between groups were made using the student-*t* test. Skewed measures were expressed as median (range), and comparisons of count data were made using the *X*^2 test or Fisher's exact probability method.

Dice score was used to evaluate the pancreatic lesion segmentation model. Accuracy, sensitivity, and specificity of the test dataset results were calculated using receiver operator characteristic curve (ROC) for the classification models. *p* values less than 0.05 were considered statistically significant.

## Results

### Patient baseline characteristics

From January 2016 to September 2021, 613 consecutive patients with PDAC were retrospectively recruited to our cancer center. Of these, 370 patients (164 women and 206 men; mean age 60.08 ± 9.36 years) were finally screened. These patients were divided into two cohorts based on pathological grading. There were 190 cases in the LG group and 180 cases in the HG group. Table 1 summarizes the baseline characteristics of the patients in the LG and HG groups.

**Table 1:** Baseline characteristics of patients

Variable	Low grade (*n* = 190)	High grade (*n* = 180)	*p* value
*Demographics*			
Age (years), mean ± SD	59.90 ± 9.22	60.28 ± 9.52	0.698
Gender			0.066
Female	93 (49%)	71 (39%)	
Male	97 (51%)	109 (61%)	
BMI^a^	23.4 (21.47–25.9)	23.44 (22.03–25.13)	0.449
Smoke	59 (31%)	60 (33%)	0.639
Drink alcohol	45 (24%)	60 (33%)	0.04
Abdominal discomfort	88 (46%)	99 (55%)	0.095
Weight loss(> 5 kg)	39 (21%)	57 (30%)	0.015
*PET/CT parameters*			
Tumor size (mm)^a^			
Max	31.5 (26.0–45.0)	31 (27.0–42.0)	0.728
Min	26 (20.0–35.5)	25 (19.5–33.0)	0.254
Tumor SUVmax^a^	5.75 (4.4–7.95)	7.5 (5.6–9.6)	0.001
Liver SUVmean	2.30 (2.02–2.54)	2.30 (2.02–2.54)	0.974
SUVR	2.74 (1.60–3.01)	3.48 (2.27–4.11)	0.003
Location			0.01
Head–neck	103 (54%)	121 (67%)	
Body–tail	87 (46%)	59 (33%)	
*Pathological report*			
Neuroaggression	113 (59%)	109 (61%)	0.832
Cancer embolus	15 (8%)	38 (21%)	0.001
Lymph node metastasis	58 (31%)	58 (32%)	0.834
*Laboratory findings*^a^			
ALT(U/L)	22.2 (11.5–58.7)	17.2 (13.2–98.5)	0.159
AST(U/L)	17.70 (13.5–49.3)	18.75 (13.5–50.8)	0.369
Total bilirubin(umol/L)	12.4 (8.7–18.6)	15.7 (8.7–32.7)	0.007
Direct bilirubin(umol/L)	4.4 (2.9–8.8)	4.9 (2.7–20.5)	0.037
Glucose (mmol/L)	5.97 (5.2–7.0)	5.48 (4.8–6.9)	0.162
CEA (μg/L)	2.77 (1.8–5.0)	3.02 (2.0–4.7)	0.325
CA125(U/mL)	14.46 (8.7–25.4)	18.96 (11.7–34.7)	0.004
CA199(U/mL)	170.25 (47.8–611.2)	229 (70.5–738.4)	0.027

^a^Data in parentheses are the interquartile range

BMI, Body Mass Index; ALT, alanine aminotransferase; AST, aspartate amino transferase; CEA, Carcinoma Embryonic Antigen; CA 125, carbohydrate antigen 125; CA 199, carbohydrate antigen 199

No significant differences in clinical characteristics were observed between these two cohorts. Alcohol consumption rates were 24.0% (45/190) and 33.3% (60/180), respectively, with a significant difference between the two cohorts (*p* = 0.04). A total of 21.0% (39 out of 190) of the LG group and 30% (87 out of 180) of the HG group had significant weight loss, with a statistically significant difference between the two groups (*p* = 0.015).

All PET/CT parameters were independently reviewed and assessed by two experienced PET/CT diagnosticians. The median value of SUVmax was 5.75 (2.2–36.0) in the LG group and 7.5 (3.2–31.1) in the HG group, with a statistically significant difference between the two groups (*p* = 0.001). There was no significant difference in liver SUVmean between the LG group and the HG group (2.30 vs. 2.30, *p* = 0.974). However, the comparison of SUVR between the two groups (2.74 vs. 3.48, *p* = 0.003) demonstrated a high level of consistency with the comparison of SUVmax between the groups, revealing a statistically significant difference. Tumors in the HG group were more likely to be found in the head and neck of the pancreas than in the LG group 67% versus 54% (*p* = 0.01), a statistically significant difference between the two groups. In laboratory tests, total and direct bilirubin levels were lower in the LG group than in the HG group (12.4 vs. 15.7, *p* = 0.007; 4.4 vs. 4.9, *p* = 0.037), with a statistically significant difference between the two groups. SUVmax is considerably influenced by blood glucose levels at the time of imaging; however, no statistically significant difference was observed in blood sugar levels between the LG and HG groups (5.97 vs. 5.48, *p* = 0.162). In terms of tumor marker detection, the LG group had lower levels of CA-125 and CA199 than the HG group (14.46 vs. 18.96, *p* = 0.004; 170.25 vs. 229, *p* = 0.027).

### Performance of lesion segmentation model

**Fig. 3:**
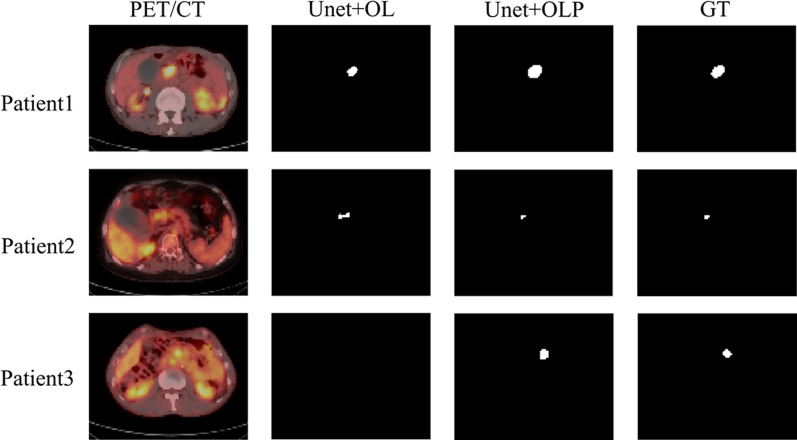
Compare the output of different segmentation models (Unet + OL: direct Unet prediction with guidance of organ location; Unet + OLP: Unet + OL prediction with post-processing; GT: Ground Truth)

The Dice score for the lesion segmentation Unet model in Additional file 1: Table S1 is 0.72. The Dice score for Unet prediction with guidance of organ location (Unet + OL) was increased to 0.76 with the addition of nnUnet-based organ segmentation. The Dice score for Unet + OL prediction with post-processing (Unet + OLP), which is Unet + OL with the addition of post-processing such as erosion, expansion, and threshold segmentation, was improved to 0.89. It is a significant advantage compared to the Unet and Unet + OL models. As shown in Fig. 3, in the three validated cases, the region of lesions output by Unet + OLP was closer to the Ground Truth (GT) labeling than that of Unet + OL.

### Performance of PDAC pathological grade classification model

**Fig. 4:**
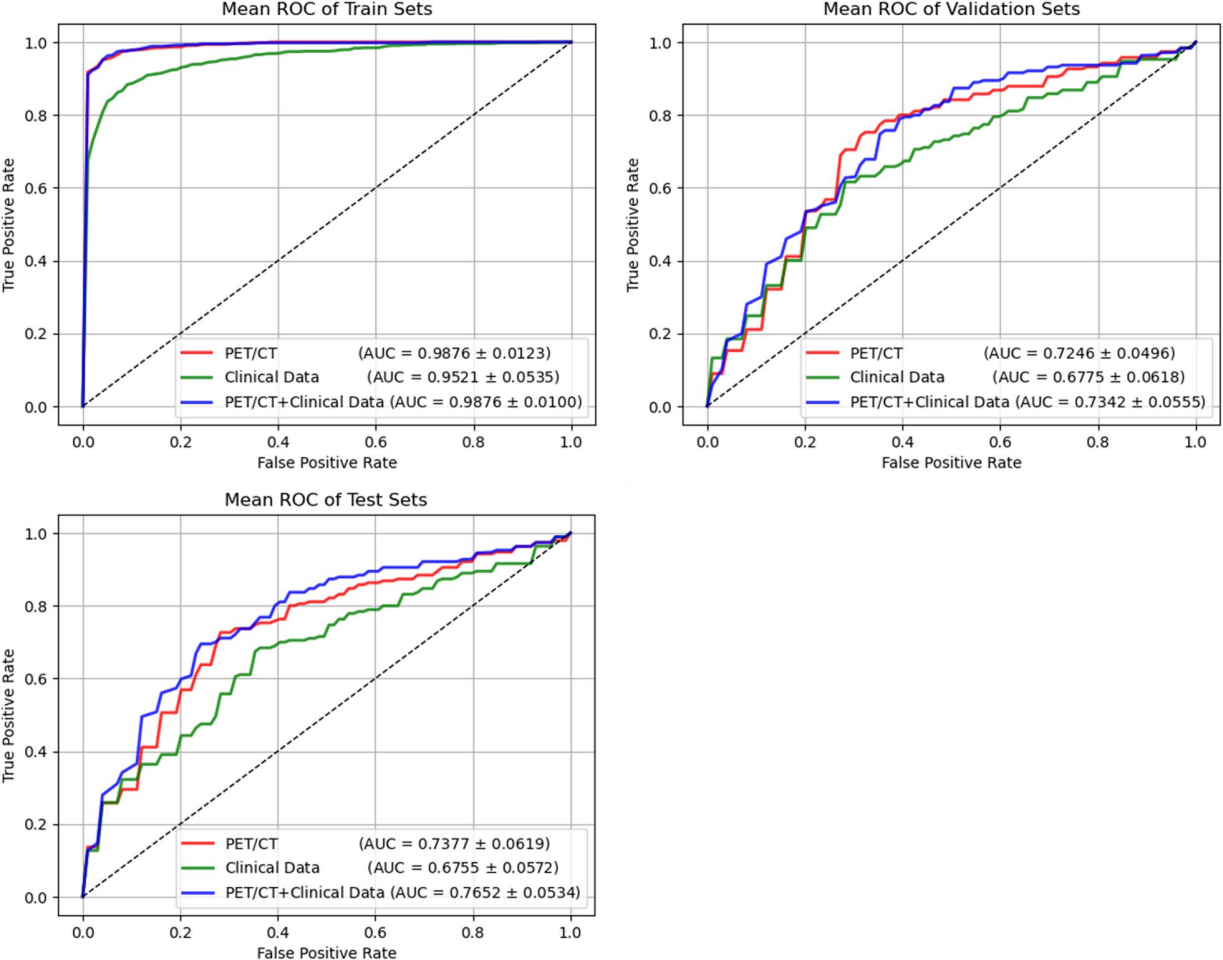
Receiver operating characteristic (ROC) curve comparison among different models for predicting the pathological grade of PDAC

Regarding testing performance, Fig. 4 shows that the AUC of the clinical data model was 0.95 in the training cohort, 0.68 in the validation cohort and 0.68 in the test cohort, while the AUC of the PET/CT model was 0.99 in the training cohort, 0.72 in the validation cohort and 0.74 in the test cohort, better than the clinical model. In order to improve the efficacy and accuracy of the model, we combined the clinical model with the PET/CT DL model to build a PET/CT + Clinical data model. The AUC of the PET/CT + Clinical data model reached 0.99, 0.74, and 0.77 in the training, validation, and test cohorts, respectively.

It is shown in Table 2 that the accuracy, sensitivity, and specificity of the PET/CT model were 72%, 73%, and 72%, respectively. The accuracy, sensitivity, and specificity of the clinical data model were 66%, 67%, and 66%, respectively. The accuracy, sensitivity, and specificity of PET/CT + Clinical data model were 75%, 77%, and 73%, respectively. The model that integrates both image and clinical data achieved the best performance.

**Table 2:** The performance comparison of different models

Models	Cohorts	AUC	ACC	SENS	SPEC	PPV	NPV
PET/CT	Train	0.99	0.97	0.97	0.97	0.97	0.96
	Val	0.72	0.73	0.72	0.74	0.75	0.70
	Test	0.74	0.72	0.73	0.72	0.72	0.72
Clinical	Train	0.95	0.91	0.90	0.91	0.92	0.89
	Val	0.68	0.67	0.66	0.69	0.75	0.59
	Test	0.68	0.66	0.67	0.66	0.69	0.63
PET/CT + Clinical	Train	0.99	0.98	0.98	0.97	0.98	0.98
	Val	0.73	0.76	0.75	0.76	0.78	0.73
	Test	**0.77**	**0.75**	**0.77**	**0.73**	**0.73**	**0.76**

AUC, area under receiver operating characteristic curve; ACC, accuracy; SENS, sensitivity; SPEC, specificity; PPV, positive predictive value; NPV, negative predictive value

In Table 3, we present the statistical analysis of AUC and ACC between different models. In the test set, the PET/CT + clinical model's AUC had a *p* value of 0.008 compared to the PET/CT model and a *p* value of 0.001 compared to the clinical data model, both demonstrating statistical significance. The ACC comparison for the PET/CT + clinical model yielded a *p* value of 0.001 when compared to the clinical data model, also indicating a statistically significant difference.

**Table 3:** Comparison between PET/CT + Clinical model and other models using significance level of Delong test for methods

Cohorts	AUC		ACC	
	PET/CT	Clinical data	PET/CT	Clinical data
Validation cohort	0.614	0.090	0.002	0.002
Test cohort	0.008	0.001	0.162	0.001

## Discussion

The aim of this retrospective study was to create a deep learning model based on PET/CT scans that could automatically analyze images without requiring manual intervention. The model was designed to categorize PDAC patients into LG and HG groups based on pathological grading. The final model achieved an AUC of 0.77, accuracy of 0.75, sensitivity of 0.77, and specificity of 0.73. As far as we know, this study is the first to use deep learning techniques to predict PDAC pathological grading, which provides a foundation for future research in this area.

Currently, research on deep learning models for PDAC mainly focuses on the disease's differential diagnosis, preoperative staging, and prognostic analysis. Wei et al. [25] used a combination of machine learning and deep learning algorithms to extract features from PET/CT images to predict the difference between PDAC and autoimmune pancreatitis, developing a multi-domain fusion model with an overall performance of AUC, accuracy, sensitivity, and specificity of 0.96, 0.90, 0.88, and 0.93, respectively. Bian et al. [26] developed and validated an automated preoperative AI algorithm for tumor and lymph node segmentation in CT imaging to predict LN metastasis in PDAC patients. Lee et al. [27] developed a deep learning model based on clinical data to predict postoperative survival in pancreatic cancer patients. The model's performance in predicting 2-year overall survival (OS) was comparable to AJCC (AUC, 0.67; *p* = 0.35), and it was better than AJCC in predicting 1-year recurrence free survival (AUC, 0.54; *p* = 0.049). Yao et al. [28] employed deep learning to examine preoperative multi-phase CT scans, developing image-based biomarkers for predicting overall survival in PDAC patients. These biomarkers can be utilized to forecast the overall survival of patients with resectable PDAC.

Research has found that the pathological grade of PDAC is largely determined by the fibrous matrix quality in its stroma. Tumors with lower differentiation have more fibrous matrix and occupy more of the contrast agent [29]. This provides a principle for pathological grading of PDAC through imaging studies. For example, Tikhonova et al. [30] used a machine learning algorithm to establish a diagnostic model for image-based PDAC grading based on preoperative CT, using data from 91 patients to establish a diagnostic model. The AUC for pathological grading ≥ 2 (or 3) was 0.75 (or 0.66). Na et al. [29] developed and validated a radiological feature based on contrast-enhanced computed tomography for preoperative prediction of histological grading of PDAC, and the AUC of the final validation set was 0.770.

The above studies are all based on CT. PET/CT, integrating PET and CT into the same device and program, characterizes the lesion from different aspects and provides metabolic information from the former and detailed anatomical information from the latter, which makes the PET/CT image to have both good clarity and a strong ability to distinguish between lesion tissue and normal tissue [31]. Some research has shown that, based on 102 patients with histologically confirmed PDAC, FDG uptake is related to the invasiveness of pancreatic cancer, and SUVmax is significantly related to pathological grading [32]. Therefore, our goal is to unleash the potential of PET/CT in pathological grading of PDAC.

In this study, in order to achieve the prediction of fully automatic PDAC pathological grading and reduce the impact of confounding factors in PET/CT images as much as possible, we first developed a deep learning model for PDAC lesion segmentation. Due to the presence of FDG uptake in organs such as the liver, adrenal gland, small intestine, and bladder in addition to the pancreatic lesion, the model showed abnormal segmentation in the initial training (Additional file 1: Figure S5). In order to increase the accuracy of segmentation, nnUnet's organ segmentation pre-trained model was added to provide a rough segmentation of abdominal organs, which are filtered out to enhance the performance of pancreatic lesion segmentation. Although nnUnet [33] is an excellent image segmentation model that demonstrated good performance in the field of medical image segmentation, especially with a large amount of training images, it is difficult to directly obtain a good segmentation model due to the small number of pancreatic cancer sections in this study. We used a pre-trained nnUnet to predict the CT part to obtain pancreas segmentation [21], preserved the parameters of the model, and applied it to the target cases. The presence of tumors greatly reduced the segmentation results of the nnUnet model, but the preliminary localization of the patient's pancreas can still be obtained. The addition of nnUnet as a filter increased the Dice score of the model from 0.72 to 0.76. In the post-processing, we added corrosion, expansion, SUVmax 40% threshold segmentation and other post-operations, and finally increased the Dice score to 0.86 (Additional file 1: Table S1.). Through these steps, the segmentation model's performance was able to achieve an acceptable level.

In building a classification model for PET/CT images, to incorporate more effective information while minimizing the effect of segmentation errors, we cropped out a 3D patch from the raw image centered around the segmented mask. The accuracy of the final PET/CT-based classification model was 72%, sensitivity was 73%, and specificity was 72%.

To further boost the classification performance, we resorted to clinical indicators that are important for revealing pancreatic cancer characteristics. For example, CA199 is closely related to the prognosis of pancreatic cancer patients [34, 35]. A study found that CA199 produced by pancreatic cancer cell lines in vitro is associated with histological differentiation in nude mice in vivo [36]. Therefore, we extracted key clinical data indicators such as CA199 and combined them with the PET/CT model to optimize the prediction accuracy. In our early experiment, we used fully connected layers to connect PET/CT with the extracted clinical features and achieved a minor performance improvement; in some folds in cross-validation, the combined model performed worser than the original PET/CT model. Therefore, in order to better integrate the features of PET/CT and clinical data, we used TMC [24] to improve the reliability of classification. This model parameterizes different data and combines them based on Dempster-Shafer theory, which improves the reliability and robustness of the classification model and improves the performance of the model (refer to Additional file 1: Method 1.2). Finally, to test the generalization ability of the model, we utilized sevenfold cross-validation. The final PET/CT + Clinical data model achieved an accuracy of 75%, sensitivity of 77%, and specificity of 73%. The deep learning model of PET/CT + Clinical data has a significant improvement compared to the traditional EUS-FNB [10] with an accuracy of 56% and sensitivity of 41%.

Our research has a few limitations. Firstly, our data are from a single center and lack external datasets for validation and evaluation. Three different scanners were used in our study. The measurement of metabolic parameters may be different due to machine parameters and ^18^F-FDG injection dose. To address this potential problem, our results were validated using SUVR. The results showed a high degree of agreement between SUVmax and SUVR. Additionally, using sevenfold cross-validation ensured the stability of the final model, which partially compensated for the lack of external validation sets. Secondly, all the data included in this study were from surgical patients, so the pathological differentiation was concentrated; however, the data for patients with excessive malignancy was relatively scarce, which did not make the model to show more significant discriminability. Finally, this study only explored the relationship between imaging features and pathological differentiation of PDAC but did not investigate the survival outcome of the patients, which is more concerned by patients and surgeons. Further research is needed to study the survival of patients.

Our developed deep learning model can automatically analyze PET/CT images without human intervention, reducing subjective errors and improving the accuracy and reliability of grading. It is important to note that deep learning models cannot completely replace professional knowledge of imaging and pathology. Before using deep learning models to predict pathological grading, professional knowledge needs to be combined for interpretation and evaluation to ensure the accuracy and reliability of the results. In conclusion, deep learning-based PET/CT has a great potential in pathological grading of pancreatic cancer, and more clinical studies are needed to prove its safety and effectiveness before deep learning models can replace traditional pathological staging methods.

## Conclusions

To the best of our knowledge, this is the first report of using a DL model for preoperative prediction of PDAC pathological grading using PET/CT. The model's predictive performance was improved by combining features of PET/CT and key clinical data.

## Supplementary Information

**Additional file 1: Fig. S1.** Structure of Unet tumor segmentation network. **Fig. S2.** Distribution data of pathological differentiation degree in medical records. **Fig. S3.** Structure of PDAC pathological grade classification network. **Fig. S4.** Random forest analysis. **Fig. S5.** Examples of segmentation model before and after adding nnUnet. **Table S1.** The performance comparison of segmentation process.

## Data Availability

The relevant images and clinical data from this study are not available because they contain private patient information. However, such data can be obtained through agency approval and signed data use agreements and/or signed material transfer agreements.

## Abbreviations

^18^F-FDG: ^18^F-fluorodeoxyglucose
ACC: Accuracy
ADC: Apparent diffusion coefficient
AUC: Area under curve
CT: Computed tomography
DL: Deep learning
EUS-FNB: Endoscopic ultrasound-guided fine-needle biopsy
FC: Fully connected
GT: Ground truth
HG: High grade
LG: Low grade
LNM: Lymph node metastasis
MRI: Magnetic resonance imaging
NPV: Negative predictive value
OL: Organ location
OLP: Organ location with post-processing
OS: Overall survival
PDAC: Pancreatic ductal adenocarcinoma
PPV: Positive predictive value
PET: Positron emission tomography
ROC: Receiver operator characteristic curve
SENS: Sensitivity
SPEC: Specificity
SUV: Standardized uptake value
SUVmax: Maximum standard uptake value
SUVmean: Mean standard uptake value
SUVR: Tumor-to-normal liver standard uptake value ratio, SUVmax of the tumor/SUVmean of the normal liver parenchyma
TMC: Trusted multi-view classification
