# Supplementary material for: 18F-FDG-PET/CT-based deep learning model for fully automated prediction of pathological grading for pancreatic ductal adenocarcinoma before surgery
Source: EJNMMI Res. 2023 May 25;13:49. doi: 10.1186/s13550-023-00985-4 (PMC10212867; doi:10.1186/s13550-023-00985-4)
Supplement: Supplementary file 1 — Additional file 1: Fig. S1. Structure of Unet tumor segmentation network. Fig. S2. Distribution data of pathological differentiation degree in medical records. Fig. S3. Structure of PDAC pathological grade classification network. Fig. S4. Random forest analysis. Fig. S5. Examples of segmentation model before and after adding nnUnet. Table S1. The performance comparison of segmentation process. [file 13550_2023_985_MOESM1_ESM.docx]

Deep learning model based on ^18^F-FDG-PET/CT for fully automated prediction of pathological grading for pancreatic ductal adenocarcinoma before surgery

**Supplementary Materials**

1. **Supplementary Methods**
   1. PET/CT scan parameters
   2. Trusted Multi-view Classification Model
2. **Supplementary Figures**
   1. **Fig S1. Structure of Unet tumor segmentation network**
   2. **Fig S2.** Distribution data of pathological differentiation degree in medical records
   3. **Fig S3** Structure of PDAC pathological grade classification network
   4. **Fig S4.** Random forest analysis
   5. **Fig S5**. Examples of segmentation model before and after adding nnUnet
3. **Supplementary Tables**
   1. **Table S1**. The performance comparison of segmentation process

**1. Supplementary Methods**

**1.1 PET/CT scan parameters**

Company instruments: GE Discovery VCT/Siemens Biograph 64 PET/CT/ uMI 510 PET/CT.

Drug: 18F-FDG was synthesized by cyclotron and automatic chemical synthesizer in the First Medical Center of PLA General Hospital. The radiochemical purity was > 95%.

Imaging methods: All patients underwent fluoro18-labeled deoxyglucose (18F-FDG) PET/CT imaging of the abdomen (or trunk) before surgery. Fasting for 4~5 h before imaging. The intravenous dose of 18F-FDG was 4 to 5 MBq/kg. PET/CT imaging was performed 60 min after injection, and patients were scanned in free breathing mode (scanning range -- abdomen: diaphragmatic apex to bladder; Trunk: base of skull to upper femur).

The low dose CT parameters were voltage=120-140kV, current=100-200mAs, rotation=0.8, layer thickness=3-5mm, pitch=0.9-1. The parameters of PET included 3-dimensional mode, 2-2.5min/bed (30% overlap), 4-5 beds/person, three iterations, 21 subsets, Gaussian filter half-height width=4.0 mm.

Reconstruction method: OSEM Truex algorithm was used for image attenuation correction. CT scanning data of the same machine was used for image attenuation correction. The display matrix was 128X128, the image layer thickness was 5mm, and three kinds of coronal, sagittal and transverse images and two kinds of image fusion were used for display.

**1.2 Trusted Multi-view Classification Model**


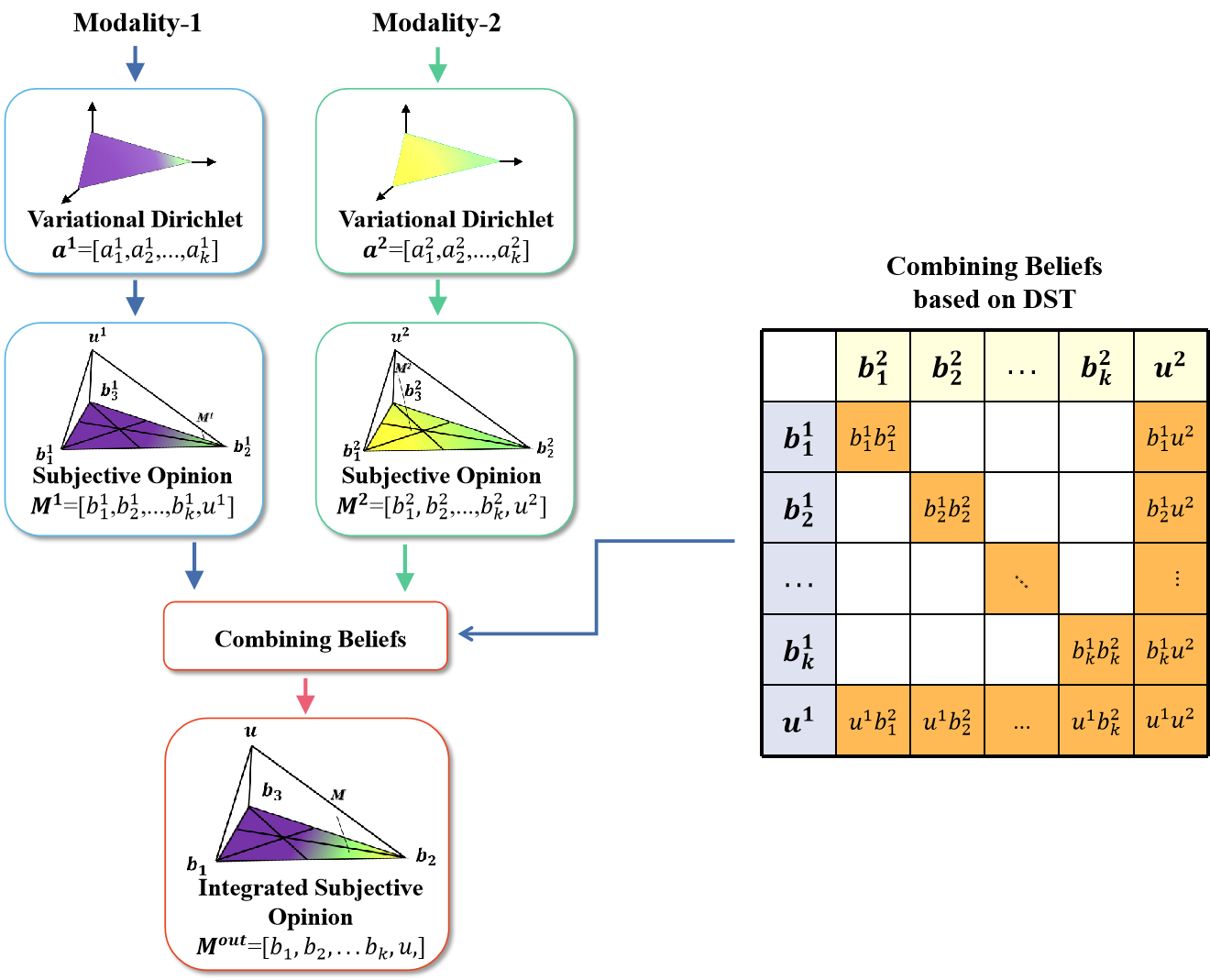


**Figure 1. Illustration of algorithm^1^**

A simplified composition of TMC algorithm is given in Fig. 1. We use two modalities as input of the model. Firstly, variational approximation is used to get the Dirichlet distribution $a^{i}$ (i=1,2) of each modality. We set modality-1 as a confident modality and modality-2 as an uncertain one. As a result, they show a completely different Dirichlet distribution map. Then, the Dirichlet distribution derives subjective opinions $M^{i}$ (i=1,2) that include beliefs and uncertainty, quantifying not only the probability of each class, but also the uncertainty of each modality. In Fig.1, M is shown as a point in a higher-dimensional space formed with beliefs **b** and uncertainty **u**. Finally, DST is adopted to integrate the subjective opinions from different modalities to obtain the combined beliefs. As a reduced rule, the compatible parts of two sets of beliefs are combined and the other ones are ignored, shown on the right part of Fig.1. After combination, it is obviously that the output is mainly effected by the confident modality-1 in our settings.

**2. Supplementary Figures**


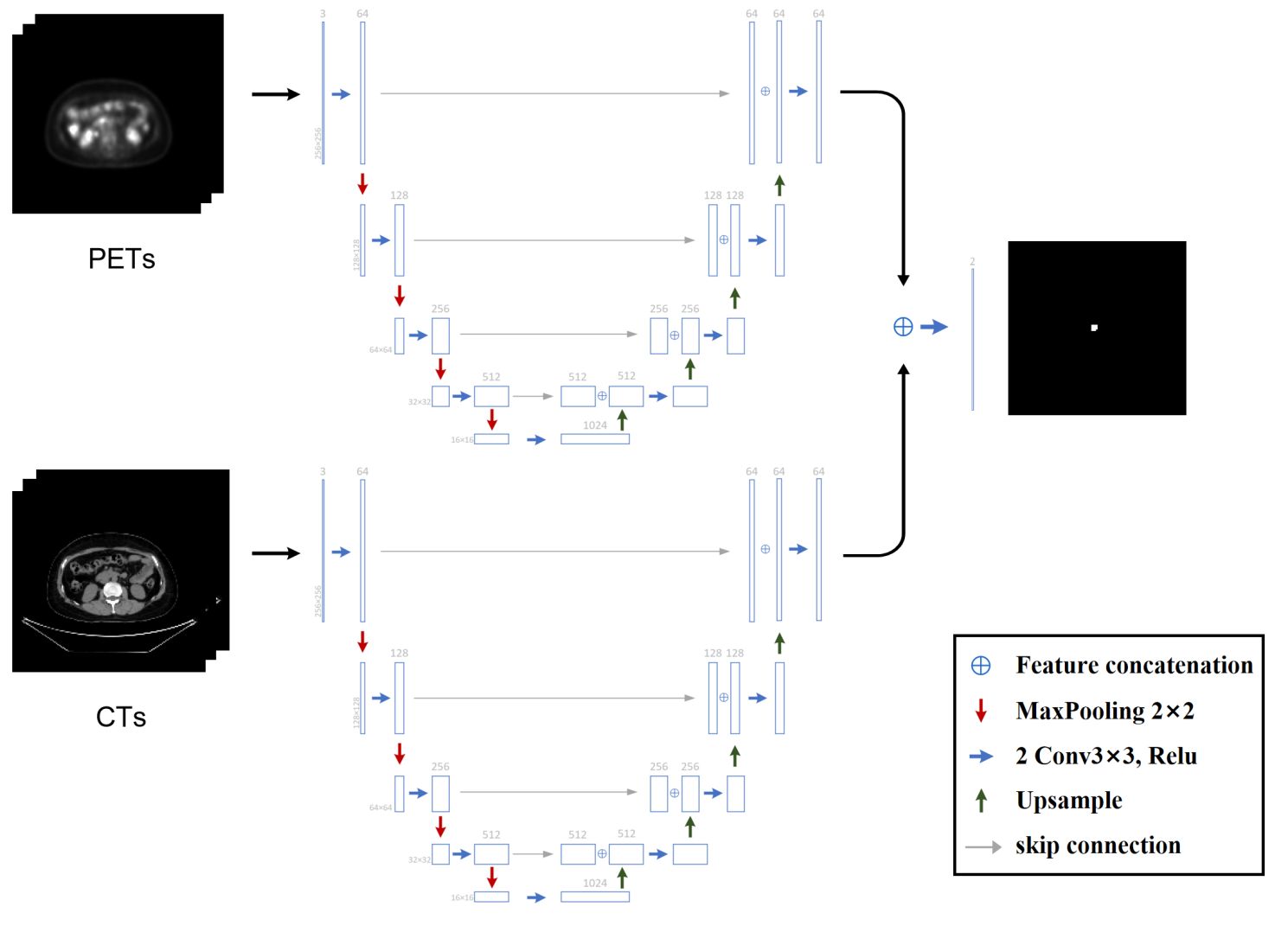


**Figure S1. Structure of Unet tumor segmentation network**


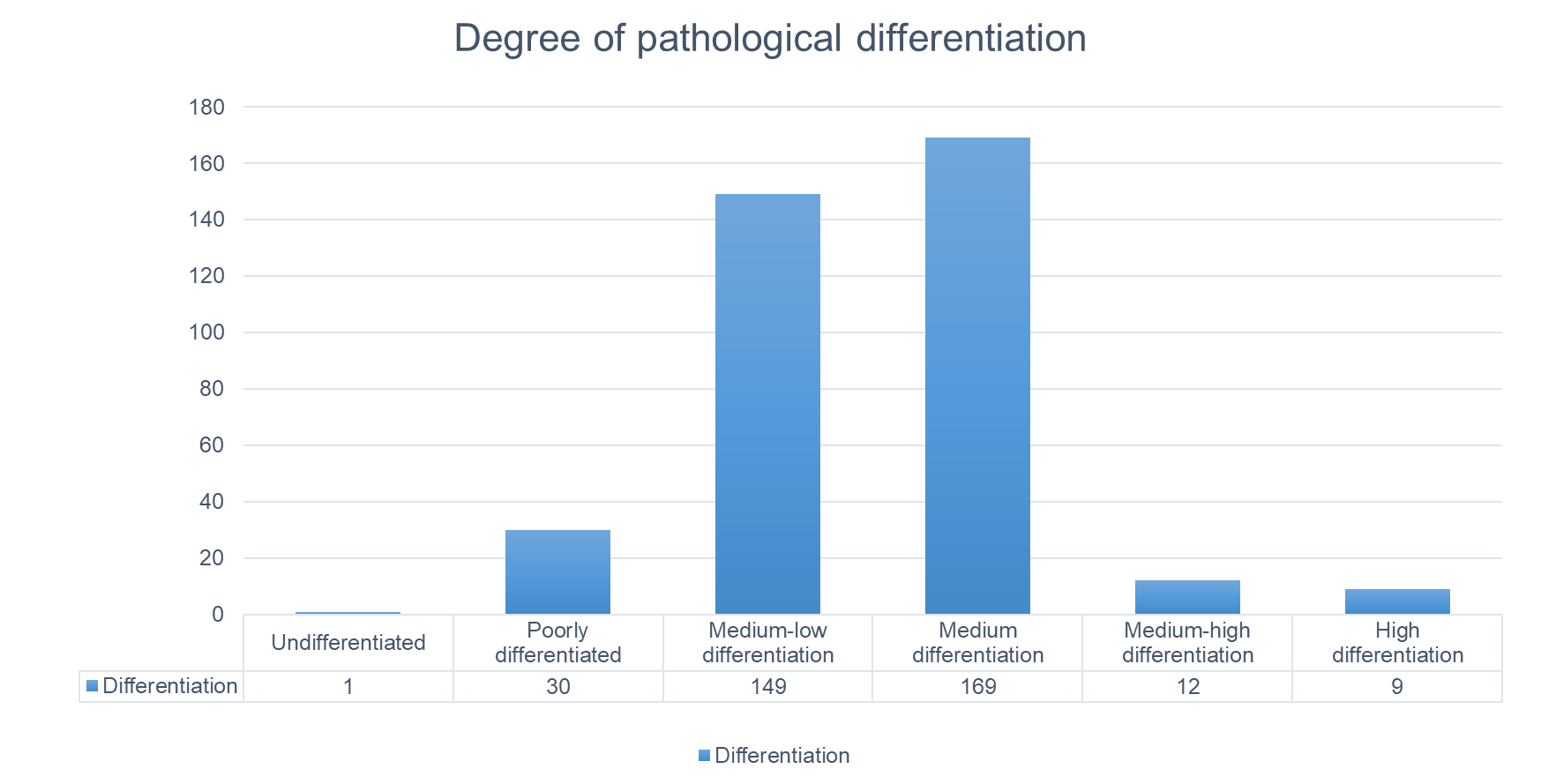


**Figure S2. Distribution data of pathological differentiation degree in medical records**


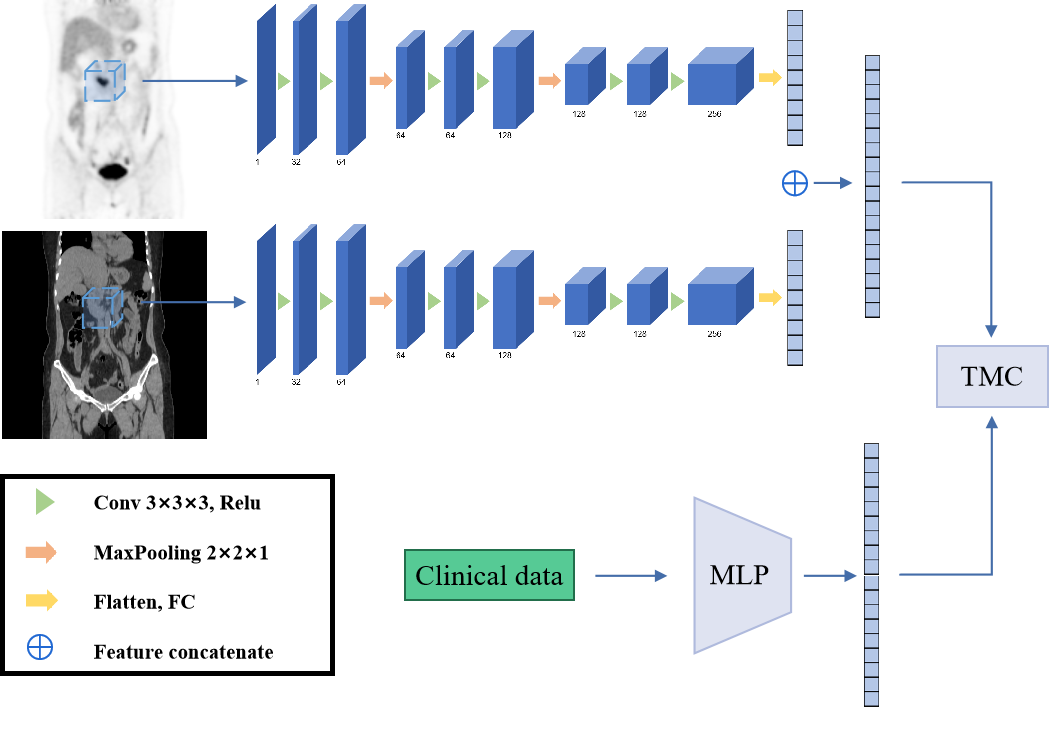


**Figure S3. Structure of PDAC pathological grade classification network**


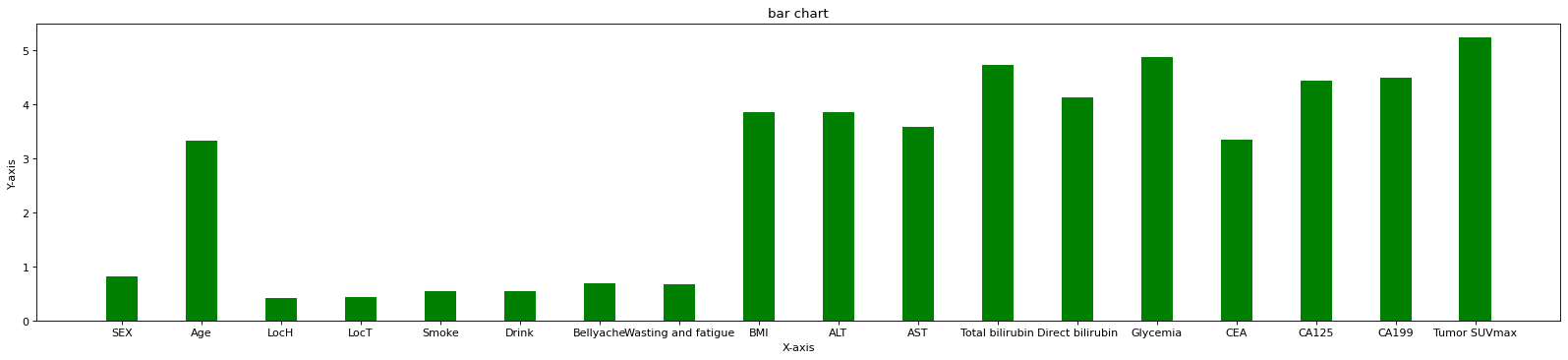


**Figure S4 Random forest analysis**

Before

After


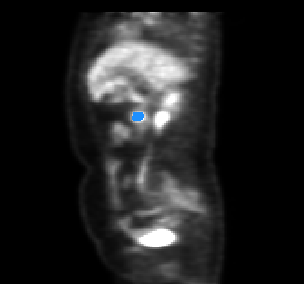

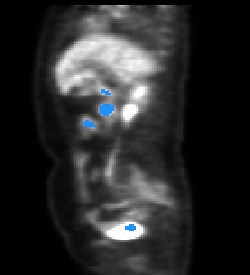


**Figure S5. Examples of segmentation model before and after adding nnUnet**

**3. Supplementary Tables**

| **Table** S1. The performance comparison of segmentation process | | | |
| --- | --- | --- | --- |
| **Models** | **Unet** | **Unet+OL** | **Unet+OLP** |
| Dice score | 0.7244 | 0.7592 | 0.8913 |
| Unet : direct Unet prediction  Unet+OL: direct Unet prediction with guidance of organ location  Unet+OLP: Unet+OL prediction with post-processing | | | |

**Reference**

1. Han Z, Zhang C, Fu H, Zhou JT. Trusted Multi-View Classification with Dynamic Evidential Fusion. *IEEE transactions on pattern analysis and machine intelligence.* 2022;Pp.
